# Clustering environmental pollutants associated with increased risk of metabolic disease: a hierarchical analysis

**DOI:** 10.1007/s13755-025-00375-1

**Published:** 2025-09-24

**Authors:** Brooke Scardino, Akshat Agrawal, Diensn G. Xing, Jackson L. St. Pierre, Md. Mostafizur Rahman Bhuiyan, Kanon Kamronnaher, Md. Shenuarin Bhuiyan, Oren Rom, Steven A. Conrad, John A. Vanchiere, A. Wayne Orr, Christopher G. Kevil, Mohammad Alfrad Nobel Bhuiyan

**Affiliations:** 1 Division of Clinical Informatics, Department of Medicine, Louisiana State University Health Sciences Center at Shreveport, PO Box 33932, Shreveport, LA 71130-3932 USA; 2 Department of Medicine, New York Institute of Technology College of Osteopathic Medicine, Jonesboro, AR 72401 USA; 3 Department of Pediatric Cardiology, Bangabandhu Sheikh Mujib Medical University, Dhaka, Bangladesh; 4 Department of Mathematical and Statistical Science, Clemson University, Clemson, SC 29634 USA; 5 Department of Pathology and Translational Pathobiology, Louisiana State University Health Sciences Center at Shreveport, Shreveport, LA 71103 USA; 6 Department of Molecular and Cellular Physiology, Louisiana State University Health Sciences Center at Shreveport, Shreveport, LA 71103 USA; 7 Department of Pharmacology, Toxicology & Neuroscience, Louisiana State University Health Sciences Center at Shreveport, Shreveport, LA 71103 USA; 8 Department of Pediatrics, Louisiana State University Health Sciences Center at Shreveport, Shreveport, LA 71103 USA

**Keywords:** Volatile organic compounds, Heavy metals, Metabolic syndrome

## Abstract

**Background:**

Metabolic syndrome (MetS), which affects one-third of the population of the United States, is a risk factor for chronic diseases such as cardiovascular diseases, stroke, and type 2 diabetes mellitus. Heavy metals (HM) and volatile organic compounds (VOC) are environmental factors typically occurring as mixtures. Although exposures to these substances have been studied separately, the impact of combined HM and VOC exposure on humans and their subsequent risk of developing MetS has not been explored. This study investigates whether combined exposure to HMs and VOCs affects the risk of developing MetS.

**Methods:**

The National Health and Nutrition Examination Survey database from 2011 to 2020 was used to determine exposure to HMs and VOCs detected in urine samples from individuals with MetS. Multiple Chi-squared and t-tests were performed to identify variables significantly associated with MetS. Logistic regression analysis was performed on unmatched and age-matched 1:1 case–control data to evaluate whether an association exists among HMs, VOCs, and demographic factors and MetS. A hierarchical cluster analysis was performed to identify combinations of HMs and VOCs linked with an increased risk of MetS.

**Results:**

Logistic regression analysis on unmatched and matched data showed that increasing age and female sex were significantly associated (*p* < 0.05) with MetS. Among the HMs and VOCs, only N-acetyl-S-(2-cyanoethyl)-l-cysteine and N-acetyl-S-(2-hydroxyethyl)-l-cysteine were found to be significantly associated with MetS. Cluster analysis showed that Cluster 3 was significantly associated with MetS (*p* < 0.05; OR = 1.49), suggesting that exposure to barium, cadmium, cesium, lead, and VOCs may increase the risk of MetS. After adjusting for covariates, none of the clusters showed a significant association (*p* > 0.05). In contrast, age (OR = 1.07) and monthly poverty level index ≤ 1.3 (OR = 1.16) were significantly associated with MetS (*p* < 0.05).

**Conclusion:**

This study revealed that age, lower socioeconomic status, and multiple exposures to combined HM and VOC may have a greater impact with an increased risk of MetS. Cluster analysis highlighted the potential combination of the exposures linked to MetS and the likelihood that demographic factors affect MetS more than exposure to HMs and VOCs. However, further research is needed.

**Supplementary Information:**

The online version contains supplementary material available at 10.1007/s13755-025-00375-1.

## Introduction

Metabolic syndrome (MetS) comprises a combination of risk factors that increase the long-term risk of diseases with significant morbidity and mortality, including cardiovascular disease (CVD), stroke, and steatotic liver disease. Currently, over one-third of population of the United States is affected by MetS, with its incidence increasing every year [1]. According to the National Cholesterol Education Program (NCEP), MetS is diagnosed when any three of the following five criteria are present: abdominal obesity, hyperlipidemia, low high-density lipoprotein cholesterol (HDL-c), hypertension (HTN), and insulin resistance [2]. A plethora of factors, individually and in combination, affect the components of MetS, with lifestyle being one of the largest effectors. Environmental toxins, such as heavy metals (HM) and volatile organic compounds (VOC), are known to have a strong relationship with MetS [3, 4]. Exposure to these substances is ubiquitous in the environment and they often enter the body through dietary intake and inhalation of polluted air.

HMs and metalloids, such as lead, cadmium, and arsenic, are indestructible and have densities greater than five times that of water [5]. Due to their higher density, these metals persist in the environment for a long time, associated with high levels of toxicity. This prolonged exposure results in accumulation within tissues, disrupting normal metabolic and cellular activities, and is positively associated with MetS [2]. This positive association between HMs and MetS might be due to overproduction of reactive oxygen species (ROS) following prolonged exposure, associated with oxidative stress, which impairs enzymatic activity and weakens antioxidant defense systems [5, 6]. Exposure to lead, commonly used in many industrial settings [1], associated with the generation of ROS that induce inflammation and promote atherosclerotic plaque buildup and clot formation [7]. Arsenic exposure is associated with obesity, the most common result of MetS in postmenopausal women [1]. Cadmium exposure increases CVD-related markers, such as C-reactive protein and low-density lipoprotein cholesterol (LDL-c) [8]. When tested in combination, exposure to HMs has been associated with higher blood pressure, lower HDL-c, and higher triglycerides, all of which are components of MetS [9].

Similarly, VOCs are low-mass, nondegradable carbon substances inhaled or ingested, mainly through smoking and carbon emissions [4]. As air quality deteriorates due to increasing emission of carbon dioxide and other air pollutants, the concentration of VOCs in the atmosphere has risen, making them a significant public health concern [4]. Once they enter the body, VOCs can bypass lung cell membranes and accumulate in the body [10]. In recent years, studies have proved an association between exposure to VOCs and MetS [11, 12].

Although these environmental exposures have been studied separately, the impact of combined exposure to HMs and VOCs remains largely unexplored. This study examines the association between MetS and combined exposure to HMs and VOCs detected in urine, using hierarchical cluster analysis on data from the National Health and Nutrition Examination Survey from 2011 to 2020.

## Materials and methods

Individuals with MetS were identified using data from the National Health and Nutrition Examination Survey (NHANES), the largest publicly available database for national cross-sectional analysis. This database encompasses patient information from 2011 to 2020, representing about 97% of Americans [13]. Each sample in the NHANES database represents over 100 clinical elements, including demographics, diagnoses, laboratory results, and clinical data for each individual patient. NHANES recorded the physical measurement data of participants at the Mobile Examination Center (MEC) [13].

MetS was classified according to the parameters defined by the NCEP [14]. Abdominal obesity was defined as a waist circumference ≥ 102 cm in men and ≥ 88 cm in women [14]. Hypertriglyceridemia was defined as serum triglyceride levels ≥ 150 mg/dL, or if the participants were receiving any medication for it [14]. Dyslipidemia was defined as serum HDL cholesterol < 40 mg/dL in men and < 50 mg/dL in women, or if the participants were receiving any medication for it [14]. Hypertension was defined as systolic blood pressure (BP) ≥ 130 mmHg or diastolic blood pressure ≥ 85 mmHg, or if the participants used antihypertensive medications [14]. Diabetes mellitus was defined as fasting plasma glucose ≥ 100 md/dl or if using anti-diabetic medicines [14].

Physical measurements and labs for serum triglycerides, HDL, and fasting glucose were taken at the MEC and recorded in approved laboratories [15]. Blood pressure was measured at the MEC by taking three consecutive measurements for each participant [16]. For this study, the third systolic and diastolic measurements were used. Medication history was obtained from the data collected using a standard questionnaire. Medications for hypertriglyceridemia and dyslipidemia were assessed if the participants were taking any cholesterol medications, antihypertensive medication was assessed if the participants were taking any blood pressure medications, and anti-diabetic medication was evaluated if the participants were taking any insulin or diabetic pills to lower blood sugar [17, 18].

Exposure to HMs and VOCs was assessed using urine samples collected at the MEC [19, 20]. Only HMs and VOCs with data available from 2011 to 2020 were included in the analysis. Urine samples were processed and analyzed at the Division of Laboratory Sciences, National Center for Environmental Health, Centers for Disease Control and Prevention in Atlanta, Georgia [19, 20].

Each of the VOCs and HMs included in this study had a specified lower limit of detection (LLOD), the values of which are included in Supplementary Tables 1 and 2. For the analytes with concentrations less than the LLOD, the value was imputed as the LLOD divided by the square root of 2 [19, 20]. The VOCs included in this study were 2-methyl hippuric acid, 3-methyl hippuric acid and 4-methyl hippuric acid, N-acetyl-S-(2-carbamoyl ethyl)-l-cysteine, N-acetyl-S-(N-methyl carbamoyl)-l-cysteine, 2-aminothiazoline-4-carboxylic acid, N-acetyl-S-(benzyl)-l-cysteine, N-acetyl-S-(n-propyl)-l-cysteine, N-acetyl-S-(2-carboxyethyl)-l-cysteine, N-acetyl-S-(2-cyanoethyl)-l-cysteine, N-acetyl-S-(3,4-dihydroxy butyl)-l-cysteine, N-acetyl-S-(2-carbamoyl-2-hydroxyethyl)-l-cysteine, N-acetyl-S-(2-hydroxyethyl)-l-cysteine, N-acetyl-S-(2-hydroxypropyl)-l-cysteine, N-acetyl-S-(3-hydroxypropyl)-l-cysteine, mandelic acid, N-acetyl-S-(4-hydroxy-2-butenyl)-l-cysteine, phenylglyoxylic acid, and N-acetyl-S-(3-hydroxypropyl-1-methyl)-l-cysteine [20]. The HMs included in the study were barium, cadmium, cobalt, cesium, molybdenum, lead, antimony, thallium, tungsten, manganese, tin, arsenic, and mercury [19].

The NHANES data are deidentified, and the Institutional Review Board (IRB) at LSU Health Sciences Center in Shreveport determined that this study is exempt from IRB oversight.

### Statistical analysis

Data from the 2011 to 2020 NHANES database was used, resulting in a final sample of 35,711 participants. The participants were stratified into different age groups for statistical analysis. Categorical variables were analyzed using a Chi-squared test, while continuous variables were assessed by t-test to evaluate the association between MetS and variables such as age, income-poverty ratio, sex, race/ethnicity, BMI, waist circumference, systolic and diastolic blood pressure, HbA1c glucose, fasting plasma glucose, HDL, LDL, triglycerides, total cholesterol, VOCs, and HMs. A logistic regression model including significant variables from the Chi-squared tests and t-tests was performed to determine which factors were associated with MetS. Variables with incomplete data were excluded from further analysis. A sensitivity analysis was performed using 1:1 case and control propensity score matching based on age, followed by logistic regression on the matched data [21].

**Fig. 1:**
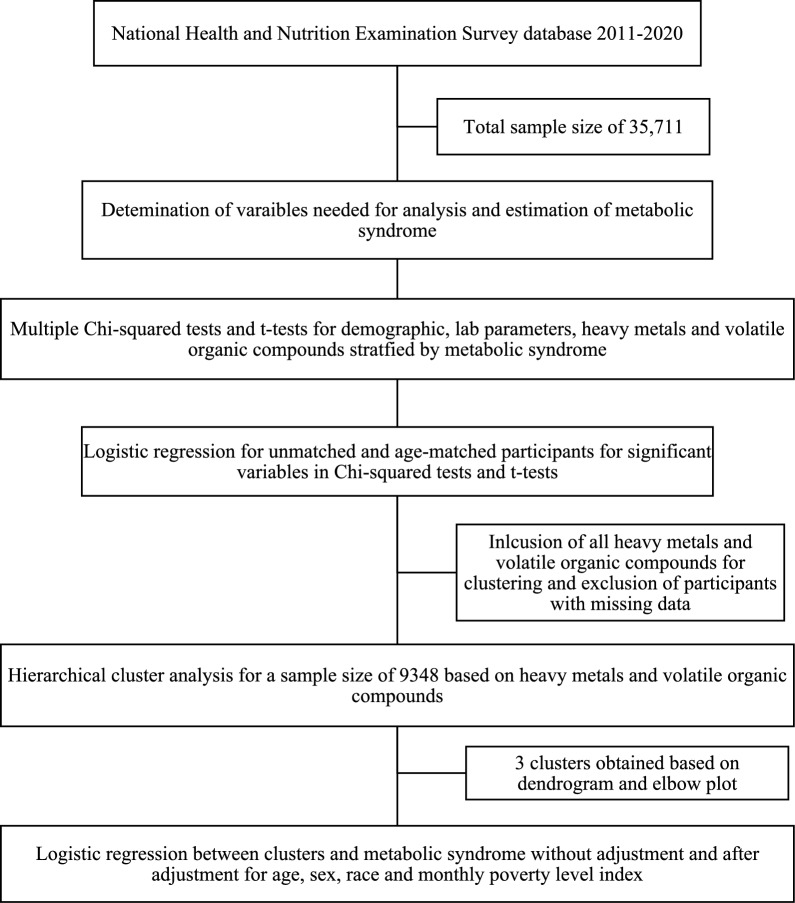
Methodology for statistical analysis of the study

The study also aimed to identify the combination of individual HMs and VOCs more likely to be associated with MetS using hierarchical clustering [22]. Hierarchical clustering analysis forms homogenous clusters by sequentially combining cases one at a time [23]. It has an advantage over other clustering methods, as it does not require a decision about the final number of clusters to be made a priori, allowing for comparison of clustering results as the number of clusters increases [23]. It also allows objective quantification of individuals based on their similarities or dissimilarities [24]. The participants were grouped into clusters based on their exposure to HMs and VOCs. All HMs and VOCs were included in hierarchical cluster analysis for better identification of clusters and any missing entries were removed for clustering for a sample size of 9348. The clusters were determined using Euclidean distances between variables and joined via Ward’s minimum variance method. A similar approach to creating clusters based on toxin exposure has been used in previous studies [24]. Using Euclidean distance and Ward’s method helps to create clusters with minimum dispersion [24]. This was followed by generation of an elbow plot and a dendrogram to identify the distinct clusters generated by the hierarchical cluster method based on HM and VOC concentrations. The plots are available in Supplementary Figs. 1 and 2. The plots show that five distinct clusters were generated. However, the last two clusters were underpowered, so the smallest three clusters were joined into one. This resulted in three unique clusters for further analysis. The mean concentrations of HMs and VOCs were calculated for each cluster, and ANOVA was used to test for differences in mean concentrations. Subsequently, a logistic regression between the identified clusters and MetS was performed to determine the cluster impact on MetS. A second logistic regression model adjusted for age, sex, race/ethnicity, and income-poverty ratio was also performed. All the statistical analyses were performed in open-source software R (version 4.4.1) [25] and the variables were considered significant for a *p* value < 0.05. The methodology used for statistical analysis in this study is shown in Fig. 1.

## Results

Data from 35,711 NHANES participants were analyzed for MetS. Nearly a quarter (8,492) met the criteria for MetS, with those affected more likely to be non-Hispanic White (hereafter White), over 65 years of age, and of lower socioeconomic status. Key markers—blood pressure, waist circumference, HDL, triglycerides, and fasting glucose—were significantly higher (*p* < 0.05) in individuals with MetS. HbA1c levels were also elevated (6.32% vs. 5.44%), as were LDL (109.7 mg/dL vs. 105.1 mg/dL) and total cholesterol (185.8 mg/dL vs. 177.8 mg/dL). Additionally, BMI was higher in those with MetS (32.53 kg/m^2^ vs. 24.31 kg/m^2^).

The average age of individuals with MetS was 58.11 years; for those without MetS, it was significantly lower (*p* < 0.05; 27.86 years). Sex was not a significant variable, but a quarter of men and women met the criteria for being diagnosed with MetS (23.38% and 24.17%, respectively). Race/ethnicity was another significant factor (*p* < 0.05) associated with MetS. White participants had the largest proportion of those with MetS (28.26%), followed by non-Hispanic Black participants (hereafter Black) (23.07%), Hispanic participants (22.36%), non-Hispanic Asian participants (hereafter Asian) (17.51%), and other/multi-racial participants (16.94%). Income also impacted the incidence of MetS. Over half of the individuals in the study population with a monthly poverty index level of 1.30–1.85 or > 1.85 had MetS (25.93% and 25.70%, respectively). Only one-fifth (20.53%) of the individuals with a monthly poverty index level < 1.30 had MetS.

HM concentrations significantly differed (*p* < 0.05) in those with MetS versus those without MetS. Cadmium and lead levels were higher in participants with MetS (0.41 and 0.53 µg/dL, respectively) than in those without MetS (0.20 µg/dL and 0.45 µg/dL, respectively). In contrast, barium (1.85 µg/dL), molybdenum (60.71 µg/dL), antimony (0.08 µg/dL), thallium (0.20 µg/dL), and tungsten (0.16 µg/dL) were lower in participants with MetS. The urinary concentrations of several VOCs was significantly higher (*p* < 0.05) in those with MetS. Concentrations of N-acetyl-S-(N-methylcarbamoyl)-l-cysteine (274.79 ng/mL), N-acetyl-S-(2-carboxyethyl)-l-cysteine (177.80 ng/mL), N-acetyl-S-(3,4-dihydroxy butyl)-l-cysteine (404.88 ng/mL), and N-acetyl-S- (4-hydroxy-2-butenyl)-l-cysteine (12.31 ng/mL) were higher in the MetS group. Only two VOCs, 2-aminothiazoline-4-carboxylic acid (240.13 ng/mL) and N-acetyl-S-(2-hydroxyethyl)-l-cysteine (1.77 ng/mL), were significantly lower (*p* < 0.05) in the MetS group. The baseline characteristics are available in Table 1.

**Table 1:** Baseline characteristics of study participants describing demographic and mean concentrations of heavy metals and volatile organic compounds stratified by metabolic syndrome

	Without metabolic syndrome	With metabolic syndrome	*p* value
Demographics			
Age (in years)	27.86	58.11	< 0.001
Sex			
Male	13,486	4115	0.08
Female	13,733	4377	
Race/ethnicity			
Hispanic	7115	2049	< 0.001
Non-Hispanic Asian	3044	646	–
Non-Hispanic Black	6800	2039	–
Non-Hispanic White	8769	3454	–
Other race	1491	304	–
BMI (kg/m^2^)	24.31	32.53	< 0.001
Waist circumference (cm)	82.52	108.96	< 0.001
Systolic BP (mmHg)	114.67	130.55	< 0.001
Diastolic BP (mmHg)	65.80	72.14	< 0.001
Income			
Monthly poverty level index ≤ 1.30	10,528	2727	< 0.001
Monthly poverty level index 1.3–1.85	3563	1247	
Monthly poverty level index > 1.85	11,277	3900	
Don’t know	519	173	
Refused	153	59	
LABS			
HbA1c (%)	5.43	6.32	< 0.001
Fasting plasma glucose (mg/dL)	97.95	124.22	< 0.001
HDL (mg/dL)	55.46	47.80	< 0.001
LDL (mg/dL)	105.12	109.70	< 0.001
Triglycerides (mg/dL)	82.70	150.93	< 0.001
Total cholesterol (mg/dL)	177.75	185.79	< 0.001
Compounds in urine			
Heavy metals (µg/L)			
Barium	1.85	1.62	< 0.001
Cadmium	0.20	0.41	< 0.001
Cobalt	0.58	0.55	0.32
Cesium	4.97	5.04	0.34
Molybdenum	60.71	49.07	< 0.001
Lead	0.45	0.53	< 0.001
Antimony	0.08	0.07	< 0.001
Thallium	0.20	0.19	< 0.001
Tungsten	0.16	0.11	< 0.001
Manganese	0.17	0.16	0.73
Tin	1.46	1.60	0.17
Arsenic	14.33	17.60	< 0.001
Mercury	0.38	0.48	0.01
Volatile organic compounds (ng/mL)			
2-Methyl hippuric acid	64.80	67.52	0.79
3-Methyl hippuric acid and 4-methyl hippuric acid	449.66	415.02	0.64
N-Acetyl-S-(2-carbamoyl ethyl)-l-cysteine	86.52	81.85	0.08
N-Acetyl-S-(N-methyl carbamoyl)-l-cysteine	170.65	274.79	< 0.001
2-Aminothiazoline-4-carboxylic acid	240.13	173.58	< 0.001
N-Acetyl-S-(benzyl)-l-cysteine	13.27	12.99	0.69
N-Acetyl-S-(n-propyl)-l-cysteine	10.94	11.22	0.68
N-Acetyl-S-(2-carboxyethyl)-l-cysteine	135.42	177.80	< 0.001
N-Acetyl-S-(2-cyanoethyl)-l-cysteine	33.31	41.05	< 0.001
N-Acetyl-S-(3,4-dihydroxy butyl)-l-cysteine	378.58	404.88	< 0.001
N-Acetyl-S-(2-carbamoyl-2-hydroxyethyl)-l-cysteine	12.76	13.05	0.38
N-Acetyl-S-(2-hydroxyethyl)-l-cysteine	1.77	1.48	< 0.001
N-Acetyl-S-(2-hydroxypropyl)-l-cysteine	62.66	71.59	0.13
N-Acetyl-S-(3-hydroxypropyl)-l-cysteine	442.35	503.70	< 0.001
Mandelic acid	176.08	203.25	0.04
N-Acetyl-S-(4-hydroxy-2-butenyl)-l-cysteine	10.25	12.31	< 0.001
Phenylglyoxylic acid	255.14	299.35	0.01
N-Acetyl-S-(3-hydroxypropyl-1-methyl)-l-cysteine	415.72	516.48	< 0.001

In the unmatched logistic regression, each participant was compared against a common standard, a Hispanic male > 60 years old, to determine which factors correlated with MetS. Unless stated otherwise, all values are considered statistically significant (*p* < 0.05). As the age of participants increased, the odds ratio increased from 0.09 in the < 18 years age group to 0.44 in the 40–59 years age group. Women were 2.80 times more likely to develop MetS. Race/ethnicity and income were not statistically significant (*p* > 0.05). Almost all hallmarks of MetS were correlated with MetS with the following adjusted odds ratios: waist circumference (OR = 4.37), systolic BP (OR = 1.64), diastolic BP (OR = 1.19), fasting plasma glucose (OR = 4.05), and triglycerides (OR = 4.87). HDL and LDL had adjusted odds ratios of 0.59 and 0.67, respectively. No HM alone exhibited statistical significance. Of the VOCs, N-acetyl-S-(2-cyanoethyl)-l-cysteine and N-acetyl-S-(2-hydroxyethyl)-l-cysteine were the only compounds significantly correlated with MetS, with adjusted odds ratios of 1.32 and 0.82, respectively.

Data were further analyzed using propensity matching, comparing participants with MetS against similarly aged participants to increase the accuracy of the results. In terms of age, only participants in the age groups 18–39 years and 40–59 years had significant results (*p* < 0.05). Their adjusted odds ratios were 0.29 and 0.46, respectively, showing that the correlation between age and MetS became stronger as age increased. Women still had a significantly higher adjusted odds ratio (*p* < 0.05) of 2.95. Race/ethnicity and income were not factors significantly correlated with MetS. The same hallmarks of MetS remained significant (*p* < 0.05): waist circumference (OR = 3.06), systolic BP (OR = 1.55), diastolic BP (OR = 1.16), fasting plasma glucose (OR = 7.14), and triglycerides (OR = 2.85). HDL and LDL were both significant (*p* < 0.05) with adjusted odds ratios of 0.63 and 0.65, respectively. HMs had no significant individual correlations to MetS. N-Acetyl-S-(2-cyanoethyl)-l-cysteine and N-acetyl-S-(2-hydroxyethyl)-l-cysteine were the only VOCs with statistically significant (*p* < 0.05) correlations to MetS, with adjusted odds ratios of 1.43 and 0.83, respectively. The results of logistic regression and propensity score matching are available in Table 2.

**Table 2:** Logistic regression analysis for unmatched participants and age matched participants using propensity score matching

Group	Variable	Unmatched		Matched	
		Adjusted odds ratio	*p* value	Adjusted odds ratio	*p* value
Age group					
< 18 years	0.09	< 0.01	1.08	0.86	
18–39 years	0.15	< 0.01	0.29	< 0.01	
40–59 years	0.44	< 0.01	0.46	< 0.01	
Sex					
Female		2.80	< 0.01	2.95	< 0.01
Race/ethnicity					
Non-Hispanic Asian	0.66	0.09	0.69	0.14	
Non-Hispanic Black	0.95	0.77	1.11	0.59	
Non-Hispanic White	0.80	0.19	0.89	0.49	
Other race	0.75	0.36	0.78	0.46	
Income-poverty ratio					
Monthly poverty level index ≤ 1.30	0.97	0.84	0.95	0.74	
Monthly poverty level index 1.3–1.85	0.79	0.16	0.75	0.10	
BMI (kg/m^2^)		1.02	0.93	1.02	0.90
Waist circumference (cm)		4.37	< 0.01	3.06	< 0.01
Systolic blood pressure (mmHg)		1.64	< 0.01	1.55	< 0.01
Diastolic blood pressure (mmHg)		1.19	0.02	1.16	0.03
HbA1c (%)		1.27	0.08	1.34	0.06
Fasting plasma glucose (mg/dL)		4.05	< 0.01	7.14	< 0.01
HDL (mg/dL)		0.59	< 0.01	0.63	< 0.01
LDL (mg/dL)		0.67	< 0.01	0.65	< 0.01
Triglycerides (mg/dL)		4.87	< 0.01	2.85	< 0.01
Heavy metals (µg/L)					
Barium		1.01	0.85	1.00	0.98
Cadmium		1.09	0.17	1.12	0.14
Molybdenum		0.88	0.21	0.92	0.23
Lead		0.89	0.10	0.83	0.07
Antimony		0.86	0.14	0.88	0.12
Thallium		1.02	0.79	1.07	0.37
Tungsten		0.99	0.95	0.98	0.77
Arsenic		1.09	0.09	1.15	0.08
Mercury		1.06	0.38	1.11	0.39
Volatile organic compounds (ng/mL)					
N-Acetyl-S-(N-methyl carbamoyl)-l-cysteine		1.11	0.6	1.23	0.61
2-Aminothiazoline-4-carboxylic acid		0.98	0.81	0.97	0.68
N-Acetyl-S-(2-carboxyethyl)-l-cysteine		1.04	0.72	1.01	0.94
N-Acetyl-S-(2-cyanoethyl)-l-cysteine		1.32	0.01	1.43	0.01
N-Acetyl-S-(3,4-dihydroxy butyl)-l-cysteine		1.07	0.54	1.10	0.38
N-Acetyl-S-(2-hydroxyethyl)-l-cysteine		0.82	0.02	0.83	0.02
N-Acetyl-S-(2-hydroxypropyl)-l-cysteine		1.09	0.27	1.08	0.35
N-Acetyl-S-(3-hydroxypropyl)-l-cysteine		0.89	0.51	0.93	0.66
Mandelic Acid		1.02	0.94	0.97	0.78
N-Acetyl-S-(4-hydroxy-2-butenyl)-l-cysteine		0.96	0.79	1.00	0.99
Phenylglyoxylic acid		0.72	0.17	0.83	0.14
N-Acetyl-S-(3-hydroxypropyl-1-methyl)-l-cysteine		1.04	0.87	0.95	0.82

Hierarchical clustering of the participants based on exposure to HMs and VOCs resulted in three clusters. Cluster 1 had 2894 participants, Cluster 2 had 5871 participants, and Cluster 3 had 583 participants. Cluster 3 had the oldest mean age of 44.76 years, whereas Cluster 1 and Cluster 2 had a similar age distribution with a mean age of 36.5 years. Cluster 3 had more men (57.5%) than women (42.5%) compared to Cluster 1 and Cluster 2. Blacks (40.3%) and Whites (40.3%) comprised most participants in Cluster 3. Cluster 1 and Cluster 2 had a more uniform distribution between different races/ethnicities. Most participants (52.5%) in Cluster 3 had lower incomes with a monthly poverty level index of ≤ 1.30. In comparison, most participants in Cluster 1 and Cluster 2 had better incomes, with a monthly poverty level index > 1.85 (48.5% and 46.9%, respectively). Cluster 1 had the lowest concentrations for all observed HMs and VOCs. Barium (2.31 µg/L), cadmium (0.77 µg/L), cesium (6.28 µg/L), and lead (0.81 µg/L) were higher in Cluster 3, whereas molybdenum (72.29 µg/L), tin (1.82 µg/L), arsenic (20.29 µg/L), and mercury (0.51 µg/L) were higher in Cluster 2. Other HMs had similar concentrations in Cluster 2 and Cluster 3. All of the observed VOCs had significantly higher concentrations in Cluster 3 compared to Cluster 1 and Cluster 2, with Cluster 2 having a higher VOC concentration than Cluster 1. The results are shown in Table 3.

**Table 3:** Cluster characteristics and mean concentrations for heavy metals and volatile organic compounds of the clusters

Characteristics	Cluster [mean (standard deviation)]			*p* value
	Cluster 1	Cluster 2	Cluster 3	
Cluster size	2894	5871	583	
Age (in years)	36.53 (22.72)	36.52 (23.44)	44.76 (16.27)	< 0.001
Male (%)	1236 (42.7)	3150 (53.7)	335 (57.5)	< 0.001
Race/ethnicity				
Non-Hispanic Asian	342 (11.8)	654 (11.1)	11 (1.9)	< 0.001
Non-Hispanic Black	568 (19.6)	1562 (26.6)	235 (40.3)	
Non-Hispanic White	1049 (36.2)	1863 (31.7)	235 (40.3)	
Hispanic	791 (27.3)	1513 (25.8)	60 (10.3)	
Other race	144 (5.0)	279 (4.8)	42 (7.2)	
Income–poverty ratio				
Monthly poverty level index ≤ 1.30	978 (36.4)	2048 (37.8)	277 (52.5)	< 0.001
Monthly poverty level index 1.3–1.85	405 (15.1)	827 (15.3)	83 (15.7)	
Monthly poverty level index > 1.85	1304 (48.5)	2541 (46.9)	168 (31.8)	
Heavy metals (µg/L)				
Barium	0.87 (0.91)	2.24 (3.49)	2.31 (2.70)	< 0.001
Cadmium	0.11 (0.11)	0.28 (0.35)	0.77 (0.77)	< 0.001
Cobalt	0.26 (0.23)	0.72 (1.54)	0.71 (0.73)	< 0.001
Cesium	2.35 (1.16)	6.13 (3.23)	6.28 (3.30)	< 0.001
Molybdenum	23.42 (15.97)	72.29 (58.53)	64.22 (48.20)	< 0.001
Lead	0.19 (0.15)	0.58 (0.91)	0.81 (0.67)	< 0.001
Antimony	0.04 (0.03)	0.10 (0.19)	0.13 (0.60)	< 0.001
Thallium	0.10 (0.05)	0.24 (0.17)	0.24 (0.14)	< 0.001
Tungsten	0.06 (0.09)	0.19 (0.74)	0.16 (0.19)	< 0.001
Manganese	0.11 (0.05)	0.19 (0.59)	0.18 (0.25)	< 0.001
Tin	0.52 (0.77)	1.82 (4.45)	1.58 (2.53)	< 0.001
Arsenic	5.88 (8.80)	20.29 (52.25)	15.29 (24.70)	< 0.001
Mercury	0.21 (0.29)	0.51 (1.68)	0.41 (0.57)	< 0.001
Volatile organic compounds (ng/mL)				
2-Methylhippuric acid	23.88 (37.00)	59.32 (145.48)	373.87 (2606.39)	< 0.001
3-Methylhippuric acid and 4-methylhippuric acid	143.22 (323.54)	396.53 (1007.45)	2663.34 (24,382.67)	< 0.001
N-Acetyl-S-(2-carbamoyl ethyl)-l-cysteine	32.96 (33.24)	89.39 (85.71)	347.65 (364.82)	< 0.001
N-Acetyl-S-(N-methyl carbamoyl)-l-cysteine	72.64 (70.10)	188.75 (183.72)	1017.21 (1964.85)	< 0.001
2-Aminothiazoline-4-carboxylic acid	90.81 (78.98)	269.06 (264.19)	355.49 (365.90)	< 0.001
N-Acetyl-S-(benzyl)-l-cysteine	5.28 (8.82)	15.31 (27.77)	37.17 (125.86)	< 0.001
N-Acetyl-S-(n-propyl)-l-cysteine	4.31 (6.61)	14.70 (41.89)	15.44 (30.11)	< 0.001
N-Acetyl-S-(2-carboxyethyl)-l-cysteine	53.43 (45.93)	156.17 (135.28)	563.44 (433.14)	< 0.001
N-Acetyl-S-(2-cyanoethyl)-l-cysteine	6.36 (19.56)	18.89 (55.76)	379.46 (289.12)	< 0.001
N-Acetyl-S-(3,4-dihydroxy butyl)-l-cysteine	173.28 (103.40)	459.79 (250.79)	810.48 (401.09)	< 0.001
N-Acetyl-S-(2-carbamoyl-2-hydroxyethyl)-l-cysteine	7.43 (3.00)	13.21 (10.27)	38.83 (36.99)	< 0.001
N-Acetyl-S-(2-hydroxyethyl)-l-cysteine (ng/ml)	0.82 (0.85)	1.59 (1.80)	7.54 (8.57)	< 0.001
N-Acetyl-S-(2-hydroxypropyl)-l-cysteine	26.73 (67.99)	78.39 (293.80)	154.92 (162.67)	< 0.001
N-Acetyl-S-(3-hydroxypropyl)-l-cysteine	148.90 (145.81)	425.78 (412.49)	2543.19 (2059.86)	< 0.001
Mandelic acid	71.11 (49.13)	193.83 (131.41)	722.15 (1501.85)	< 0.001
N-Acetyl-S-(4-hydroxy-2-butenyl)-l-cysteine	3.13 (3.63)	9.11 (9.97)	71.23 (47.64)	< 0.001
Phenylglyoxylic acid	115.60 (74.81)	292.91 (179.91)	873.98 (1801.50)	< 0.001
N-Acetyl-S-(3-hydroxypropyl-1-methyl)-l-cysteine	141.88 (147.23)	395.08 (440.89)	2621.57 (1794.99)	< 0.001

Logistic regression analysis carried out between the clusters based on exposure to HMs and VOCs and development of MetS showed that Cluster 2 was more likely to develop MetS (*p* = 0.05) compared to Cluster 1 (OR = 1.11, CI 1.00–1.23). Cluster 3 was significantly more likely to develop MetS (*p* < 0.01) than Cluster 1 (OR = 1.49, CI 1.22–1.80). When the logistic regression analysis was performed while adjusting for covariates (age, sex, race/ethnicity, and monthly poverty level index), the clusters were not more likely to develop MetS compared to each other (*p* > 0.05). Age (OR = 1.07), participants with Hispanic ethnicity (OR = 1.34) and Blacks (OR = 1.22) compared to Whites (OR = 0.75), and monthly poverty level index ≤ 1.3 (OR = 1.16) were significantly related (*p* < 0.05) to the risk for MetS. The results can be seen in Tables 4 and 5.

**Table 4:** Logistic regression analysis for the risk of metabolic syndrome between the clusters based on heavy metals and volatile organic compound exposure

Variable	Odds ratio (confidence interval)	*p* value
Cluster 2	1.11 (1.00–1.23)	0.05
Cluster 3	1.49 (1.22–1.80)	< 0.01

**Table 5:** Logistic regression analysis for the risk of metabolic syndrome between the clusters based on heavy metals and volatile organic compound exposure adjusted for covariates

Variable	Odds ratio (confidence interval)	*p* value
Cluster 2	1.11 (0.98–1.27)	0.11
Cluster 3	1.15 (0.91–1.46)	0.24
Age (in years)	1.07 (1.07–1.07)	< 0.01
Sex		
Male	0.97 (0.86–1.09)	0.58
Race/ethnicity		
Hispanic	1.34 (1.14–1.56)	< 0.01
Non-Hispanic Asian	0.85 (0.68–1.04)	0.12
Non-Hispanic Black	1.22 (1.05–1.42)	0.01
Other race	0.98 (0.71–1.34)	0.89
Monthly poverty level index		
Monthly poverty level index ≤ 1.30	1.16 (1.02–1.33)	0.03
Monthly poverty level index 1.3–1.85	1.18 (1.00–1.4)	0.05

## Discussion

Using hierarchical cluster analysis in a nationally representative sample obtained from the NHANES database, the study evaluates and establishes the impact of combined exposure to HMs and VOCs on the development of MetS. The analysis showed that combined exposure to HMs and VOCs may have an increased risk of developing MetS; the cluster with higher HM and VOC concentrations had the highest risk. Exposure to increasing concentrations of barium, cadmium, cesium, lead, and VOCs were most significantly associated with MetS. An individual’s demographic characteristics had a significant effect on the risk of developing MetS; age, being Black or of Hispanic ethnicity, and lower socioeconomic status were associated with an increased risk of exposure to environmental toxins and MetS.

Various sources have been implicated in HM and VOC exposure; most exposure occurs when using products used in daily life, including food and water. Water sources commonly used for human and agricultural purposes are contaminated with HMs from mining activities, industrial affluents, and domestic sewage [26]. Soil used for farming also has elevated concentrations of HMs from continued used of fertilizers [26]. These HMs become bioconcentrated as they move up the food chain, eventually reaching humans in higher concentrations [26, 27]. VOC exposure can also occur from common sources, including vehicular emissions, moth repellants, chlorinated solvents, cleaning products, odorants, and cooking related activities [28].

The association among different lab biomarkers, demographics, and exposure to HMs and VOCs and the development of MetS was assessed using logistic regression, where each variable was compared to a random subject and a subject of similar age derived using propensity score matching. The odds ratio across all age groups was less than 1, indicating that MetS is not as likely in these age groups as in those participants over the age of 60. As age increases, the likelihood of developing MetS also increases due to the normal aging process. This includes weight gain and the onset of chronic diseases related to MetS, such as diabetes mellitus and heart failure [29]. Increasing age also reflects the typical age of onset for MetS comorbidities, such as type 2 diabetes mellitus and cardiovascular disease [30]. Women were also more likely than men to have MetS. This could be due to their lack of estrogen post-menopause, which is associated with increased abdominal obesity and insulin resistance [31]. Pollutants such as barium and cadmium, which affect estrogen receptors and cause impaired fat metabolism and increased insulin resistance, contribute to the pathogenesis of MetS [32]. Race/ethnicity and income alone were not enough to indicate a relationship for MetS, although individuals with MetS in lower socio-economic households face challenges, including poor lifestyle choices, limited access to healthcare, and lack of health-based education [33]. All hallmarks for MetS exhibited an odds ratio of > 1, apart from HDL, which had an odds ratio of < 1, as expected. LDL was less correlated to MetS, with an odds ratio of < 1. This finding could be caused by the association between lower LDL levels and increased triglyceride levels during MetS [34]. N-Acetyl-S-(2-cyanoethyl)-l-cysteine had a significant positive correlation with MetS. This VOC is produced after exposure to acrylonitrile, found in tobacco smoke and polymers, and is known for affecting the detoxification pathway by increasing oxidative stress, a characteristic of MetS [35]. N-Acetyl-S-(2-hydroxyethyl)-l-cysteine is an end-product of the glutathione pathway, and lower levels could be due to impaired liver detoxification in MetS [36]. None of the other HMs or VOCs showed a significant association with MetS. Using single pollutant models, previous studies have shown them to be associated with poorer health, but factoring for related pollutants revealed no specific pollutant with a statistically significant association as was seen in our study with logistic regression analysis [37]. This shows that combined exposure to environmental toxins along with demographic risk factors increases the risk of MetS, rather than exposure to a single toxin, as determined by hierarchical cluster analysis in our study.

Hierarchical cluster analysis provided essential information regarding the risk of developing MetS due to multiple and simultaneous exposure to HMs and VOCs. People having simultaneous exposure to barium, cadmium, cesium, lead, and most VOCs were at the most risk of developing MetS, as seen in Cluster 3. Exposure to molybdenum, arsenic, tin, mercury, and VOCs also slightly increased the risk, as seen in Cluster 2. This provides important information: people exposed to multiple compounds (in our study, HMs and VOCs) are at increased risk of developing MetS as opposed to those whose exposure is limited to a single compound.

It is known that exposure to HMs and VOCs individually increases the risk of developing MetS [1, 4]. Metals including cobalt, cadmium, lead, and barium increase the risk of MetS and its components. They have been shown to be associated with increased risk of obesity, high blood pressure, and increased insulin resistance, associated with impaired glucose levels [38–41]. Similarly, VOCs increase the risk of MetS and its components. VOCs have been associated with impaired glucose metabolism and obesity [11, 42]. N-Acetyl-S-(N-methyl carbamoyl)-l-cysteine and N-acetyl-S-(2-carboxyethyl)-l-cysteine are associated with impaired MetS components [12]. Various mechanisms have been proposed to explain the mechanisms responsible for MetS caused by HMs and VOCs. One of the proposed mechanisms is increased oxidative stress and damage. HMs have been shown to form complexes with enzymes and inhibit mitochondrial function. This disrupts cellular function associated with increased ROS production and contributing to oxidative damage [43, 44]. VOCs also contribute to the oxidative stress caused by oxidative DNA damage, protein adduction, heme-oxygenase activation, protein carbonylation, and mitochondrial metabolism disruption [45–47]. They are associated with increased oxidative stress biomarkers such as 8-hydroxydeoxyguanosine, 8-hydroxyguanosine, and 4-hydroxy-2-nonenal-mercapturic acid [48]. Even low levels of VOC exposure are associated with increased oxidative stress [49]. Other mechanisms attributed to HMs and VOCs include interference with insulin receptors, increased gluconeogenesis, and altered hepatic gene expression for lipid synthesis, all of which contribute to MetS [32, 44, 50]. However, none of the previous studies determined the association between MetS and combined exposure to HMs and VOCs, since they investigated the association between MetS and HMs or VOCs individually. Our study assesses the combined effect of HMs and VOCs on MetS, shows that exposure to both HMs and VOCs may potentiate the risk of developing MetS, and elucidates the combination of exposures more likely to be associated with MetS.

When adjusting the model with clusters for demographic covariates (age, race/ethnicity, sex, and monthly poverty level index), no single cluster was identified as more at risk for developing MetS. Age was significantly associated with an increased risk of exposure and developing MetS, consistent with findings from other studies [29]. Hispanic and Black participants exhibited a higher risk compared to White participants, with people having a monthly poverty level index ≤ 1.30 at the most significant risk of MetS. This suggests that economic disadvantage and exposure to HMs and VOCs are significant risk factors for the development of MetS. Prior research has shown that levels of HMs and VOCs are higher in populations living in lower-income areas, and among Hispanics and Blacks, similar to our study [51, 52]. Poor lifestyle choices, such as smoking and consumption of unhealthy diets, further increases exposure and the risk of MetS [53]. Our analysis indicated that while exposure to certain HMs and VOCs contributes to an increased risk of developing MetS, the influence of demographic factors on this risk is even more pronounced among individuals exposed to these environmental toxins.

Our study provides evidence for increased risk of MetS following cumulative exposure to HMs and VOCs in concert with demographic risk factors. Future longitudinal research designs can help establish the causality between combined exposure to toxins (HMs and VOCs) and the development of MetS in a population with varied demographic and socioeconomic backgrounds. Future studies can also collect geographical data with estimated exposure sources to perform a geospatial analysis to identify regions more at risk for exposure and subsequent development of MetS along with other diseases.

## Conclusion

This study provides new insights into the risk factors associated with developing MetS including exposure to HMs and VOCs. MetS is more prevalent in individuals with advanced age and lower socioeconomic status, with risk escalating for those exposed to multiple risk factors. Cluster analysis further illuminated the potential interaction among various compounds that may heighten the risk of MetS. Additionally, demographic covariates substantially influence the prevalence of MetS, reinforcing that social and economic factors may enhance exposure to HMs and VOCs. These findings underscore the need for public health policy reforms that address HM and VOC exposure, especially given the epidemic of obesity and MetS in the United States.

## Supplementary Information

Below is the link to the electronic supplementary material.

Supplementary file1 (DOCX 197 KB)

## Data Availability

The data used for the study are a publicly available at the National Health and Nutrition Examination Survey 2011–2020 database: https://wwwn.cdc.gov/nchs/nhanes/continuousnhanes/default.aspx?Cycle=2017-2020, https://wwwn.cdc.gov/nchs/nhanes/continuousnhanes/default.aspx?BeginYear=2015, https://wwwn.cdc.gov/nchs/nhanes/continuousnhanes/default.aspx?BeginYear=2013, and https://wwwn.cdc.gov/nchs/nhanes/continuousnhanes/default.aspx?BeginYear=2011, under the section data, documentation, and codebooks. The data were analyzed using the open-source statistical software R.

## Declarations

### Approval of the research protocol

NA.

### Registry and the registration no. of the study/trial

NA.

### Research involving animal studies

NA.

### Research involving recombinant DNA

NA.
