# Supplementary material for: Clustering environmental pollutants associated with increased risk of metabolic disease: a hierarchical analysis
Source: Health Inf Sci Syst. 2025 Sep 24;13(1):59. doi: 10.1007/s13755-025-00375-1 (PMC12460862; doi:10.1007/s13755-025-00375-1)
Supplement: Supplementary file 1 — Supplementary file1 (DOCX 197 KB) [file 13755_2025_375_MOESM1_ESM.docx]

**Clustering Environmental Pollutants Associated with Increased Risk of Metabolic Disease – A Hierarchical Analysis**

Brooke Scardino, B.S.^1#^, Akshat Agrawal, MBBS^1#^, Diensn Xing, BSc^1^, Jackson L. St. Pierre, B.S.^2^, Md. Mostafizur Rahman Bhuiyan, MD^3^, Kanon Kamronnaher, PhD^4^, Md. Shenuarin Bhuiyan, PhD^5,6^, Oren Rom, PhD^5,6^, Steven A. Conrad, MD, PhD^1,7^, John A. Vanchiere, MD, PhD^1,8^, A Wayne Orr, PhD^5,6^, Christopher G. Kevil, PhD^5,6^, Mohammad Alfrad Nobel Bhuiyan, PhD^*1,5^

^1^Department of Medicine, Louisiana State University Health Sciences Center at Shreveport, Shreveport, LA, 71103, USA

^2^Department of Medicine, New York Institute of Technology College of Osteopathic Medicine, Jonesboro, AR, 72401, USA

^3^Department of Pediatric Cardiology, Bangabandhu Sheikh Mujib Medical University, Dhaka, Bangladesh

^4^Department of Mathematical and Statistical Science, Clemson University, Clemson, SC, 29634, USA

^5^Department of Pathology and Translational Pathobiology, Louisiana State University Health Sciences Center at Shreveport, Shreveport, LA, 71103, USA

^6^Department of Molecular and Cellular Physiology, Louisiana State University Health Sciences Center at Shreveport, Shreveport, LA, 71103, USA

^7^Department of Pharmacology, Toxicology & Neuroscience, Louisiana State University Health Sciences Center at Shreveport, Shreveport, LA, 71103, USA

^8^Department of Pediatrics, Louisiana State University Health Sciences Center at Shreveport, Shreveport, LA, 71103, USA

#These authors contributed equally.

***Correspondence to:** Mohammad Alfrad Nobel Bhuiyan, PhD, Division of Clinical Informatics, Department of Medicine, Louisiana State University Health Sciences Center, PO Box 33932, Shreveport, LA 71130-3932. Email: [Nobel.Bhuiyan@lsuhs.edu](mailto:Nobel.Bhuiyan@lsuhs.edu)

**Supplement Table 1. Lower limit of detection (µg/L) for heavy metals**

| **Compound** | **Lower limit of detection [release cycle]** |
| --- | --- |
| Barium (µg/L) | 0.10 [2011-2012]  0.060 [2013-2016]  0.084 [2017-2020] |
| Cadmium (µg/L) | 0.056 [11-12]  0.036 [2013-2016]  0.055 [2017-2020] |
| Cobalt (µg/L) | 0.048 [2011-2012]  0.024 [2013-2020] |
| Cesium (µg/L) | 0.12 [2011-2012]  0.086 [2013-2016]  0.130 [2017-2020] |
| Molybdenum (µg/L) | 0.99 [2011-2012]  0.80 [2013-2020] |
| Lead (µg/L) | 0.080 [2011-2012]  0.030 [2013-2020] |
| Antimony (µg/L) | 0.0410 [2011-2012]  0.022 [2013-2020] |
| Thallium (µg/L) | 0.020 [2011-2012] |
| Tungsten (µg/L) | 0.018 [2013-2020]  0.026 [2011-2012] |
| Manganese (µg/L) | 0.080 [2011-2012]  0.130 [2013-2020] |
| Tin (µg/L) | 0.22 [2011-2012]  0.090 [2013-2016]  0.20 [2017-2020] |
| Arsenic (µg/L) | 1.25 [2011-2012]  0.26 [2013-2016]  0.23 [2017-2020] |
| Mercury (µg/L) | 0.05 [2011-2012]  0.13 [2013-2020] |

**Supplement Table 2. Lower limit of detection (ng/mL) for volatile organic compounds**

| **Compound** | **Lower limit of detection [release cycle]** |
| --- | --- |
| 2-methylhippuric acid (ng/mL) | 5.00 [2011-2020] |
| 3-methylhippuric acid and 4-methylhippuric acid (ng/mL) | 8.00 [2011-2020] |
| N-acetyl-S-(2-carbamoylethyl)-L-cysteine (ng/mL) | 2.20 [2011-2020] |
| N-acetyl-S-(N-methylcarbamoyl)-L-cysteine (ng/mL) | 6.26 [2011-2020] |
| 2-aminothiazoline-4-carboxylic acid (ng/mL) | 15 [2011-2016]  29.5 [2017-2020] |
| N-acetyl-S-(benzyl)-L-cysteine (ng/mL) | 0.50 [2011-2020] |
| N-acetyl-S-(n-propyl)-L-cysteine (ng/mL) | 1.20 [2011-2020] |
| N-acetyl-S-(2-carboxyethyl)-L-cysteine (ng/mL) | 6.96 [2011-2020] |
| N-acetyl-S-(2-cyanoethyl)-L-cysteine (ng/mL) | 0.50 [2011-2020] |
| N-acetyl-S-(3,4-dihidroxybutyl)-L-cysteine (ng/mL) | 5.25 [2011-2020] |
| N-acetyl-S-(2-carbamoyl-2-hydroxyethyl)-L-cysteine (ng/mL) | 9.40 [2011-2020] |
| N-acetyl-S-(2-hydroxyethyl)-L-cysteine (ng/mL) | 0.791 [2011-2020] |
| N-acetyl-S-(2-hydroxypropyl)-L-cysteine (ng/mL) | 5.3 [2011-2020] |
| N-acetyl-S-(3-hydroxypropyl)-L-cysteine (ng/mL) | 13.0 [2011-2020] |
| Mandelic acid (ng/mL) | 12.0 [2011-2020] |
| N-acetyl-S-(4-hydroxy-2-butenyl)-L-cysteine (ng/mL) | 0.60 [2011-2020] |
| Phenylglyoxylic acid (ng/mL) | 12.0 [2011-2020] |
| N-acetyl-S-(3-hydroxypropyl-1-methyl)-L-cysteine (ng/mL) | 1.13 [2011-2012]  1.70 [2013-2020] |

**Supplement Figure 1. Dendrogram showing cluster arrangement at various levels of similarities**

**
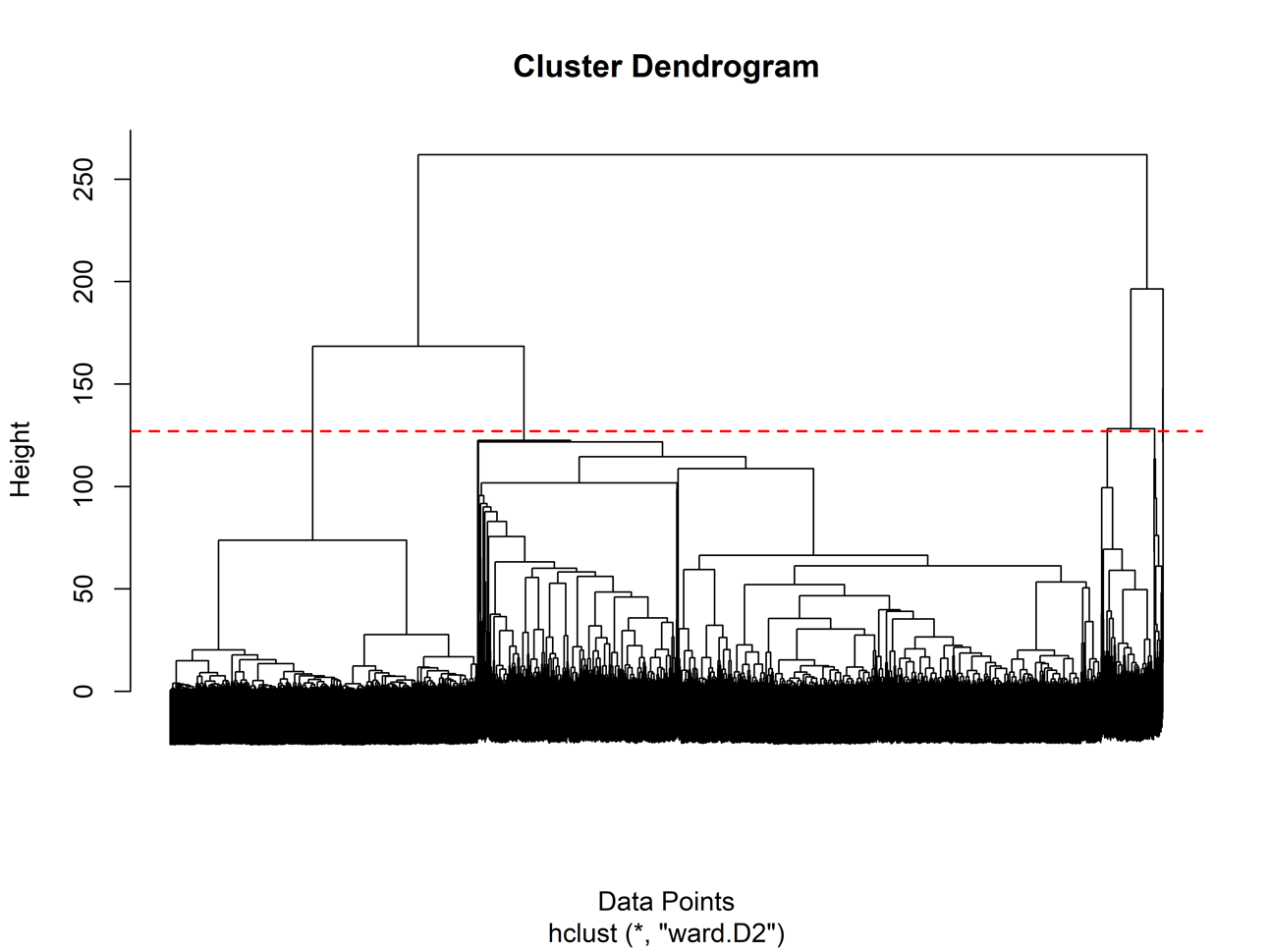
**

Note: The red horizontal line intersects the dendrogram, indicating the formation of five major clusters based on hierarchical cluster analysis.

**Supplement Figure 2. Elbow plot showing rate of change in height (distance) between the mergers**


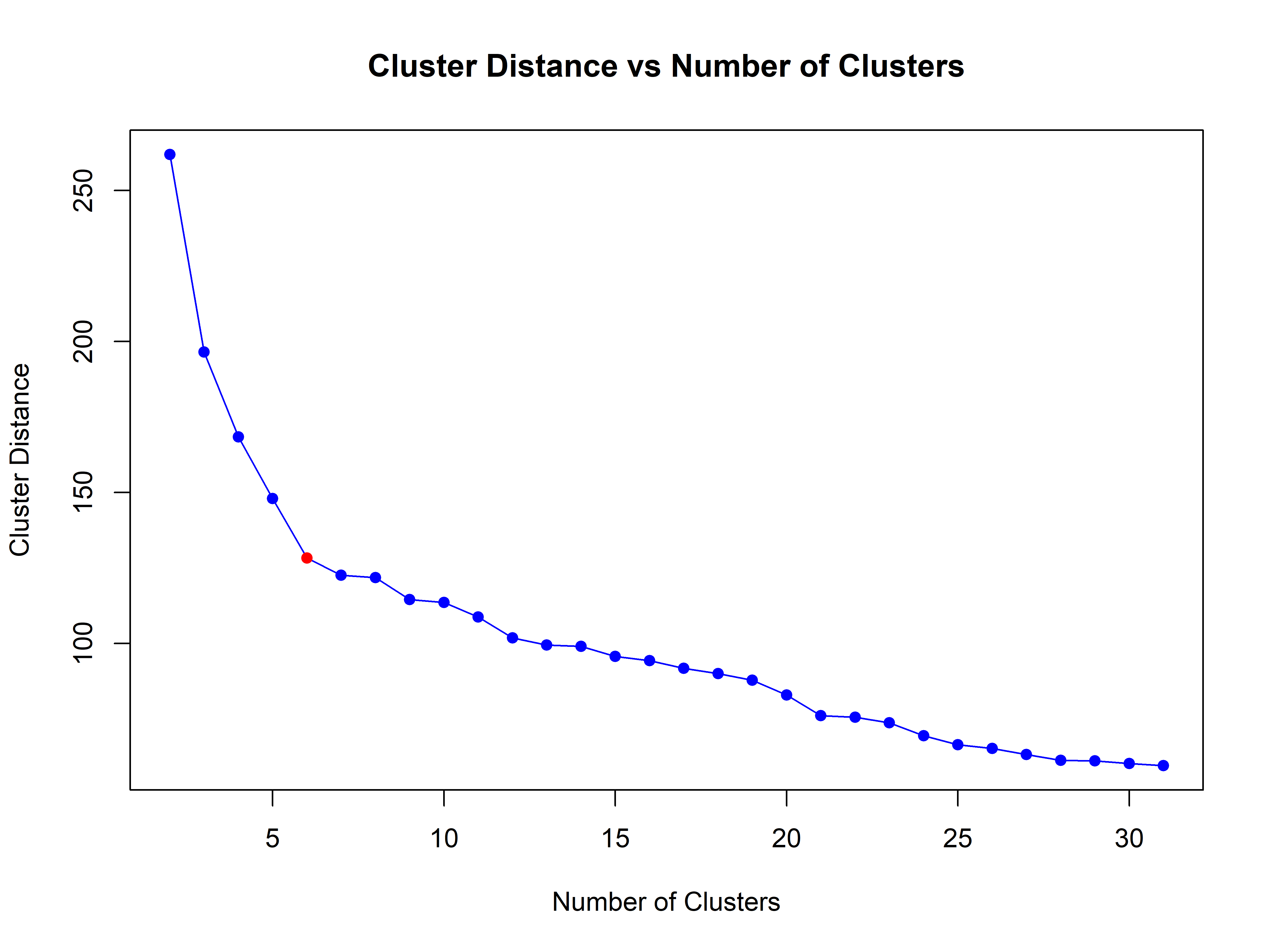
Note: The red dot in the elbow plot marks the inflection point, indicating marginal gain in clustering performance beyond five clusters, as determined by hierarchical cluster analysis.
